# Recent Advances in Bioorthogonal Click Chemistry for Efficient Synthesis of Radiotracers and Radiopharmaceuticals

**DOI:** 10.3390/molecules24193567

**Published:** 2019-10-02

**Authors:** Sajid Mushtaq, Seong-Jae Yun, Jongho Jeon

**Affiliations:** 1 Department of Nuclear Engineering, Pakistan Institute of Engineering and Applied Sciences, Islamabad 45650, Pakistan; sajidmushtaq@pieas.edu.pk; 2 IT Convergence Materials Group, Korea Institute of Industrial Technology, Cheonan 31056, Korea; ysj8570@gmail.com; 3 Department of Applied Chemistry, School of Applied Chemical Engineering, Kyungpook National University, Daegu 41566, Korea

**Keywords:** radiolabeling, bioorthogonal reaction, click chemistry, site-specific reaction, radiopharmaceuticals, radioisotopes, molecular imaging

## Abstract

In recent years, several catalyst-free site-specific reactions have been investigated for the efficient conjugation of biomolecules, nanomaterials, and living cells. Representative functional group pairs for these reactions include the following: (1) azide and cyclooctyne for strain-promoted cycloaddition reaction, (2) tetrazine and trans-alkene for inverse-electron-demand-Diels–Alder reaction, and (3) electrophilic heterocycles and cysteine for rapid condensation/addition reaction. Due to their excellent specificities and high reaction rates, these conjugation methods have been utilized for the labeling of radioisotopes (e.g., radiohalogens, radiometals) to various target molecules. The radiolabeled products prepared by these methods have been applied to preclinical research, such as in vivo molecular imaging, pharmacokinetic studies, and radiation therapy of cancer cells. In this review, we explain the basics of these chemical reactions and introduce their recent applications in the field of radiopharmacy and chemical biology. In addition, we discuss the significance, current challenges, and prospects of using bioorthogonal conjugation reactions.

## 1. Introduction

**Figure 1:**
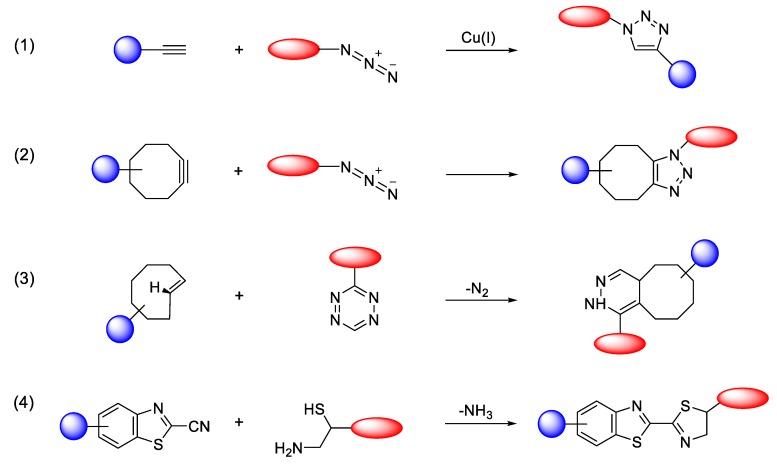
Selected bioorthogonal conjugation reactions. (**1**) Copper-catalyzed azide-alkyne cycloaddition reaction (CuAAC); (**2**) strain-promoted azide-alkyne cycloaddition reaction (SPAAC); (**3**) tetrazine and trans-alkene substrates for inverse electron-demand-Diels–Alder reaction (IEDDA); (**4**) condensation reaction between 2-cyanobenzothiazole (CBT) and 1,2-aminothiol (*N*-terminal cysteine).

The term ‘click chemistry’ has been introduced to describe specific chemical reactions, which are fast, reliable and can be selectively applied to the synthesis of functional materials and biomolecule conjugates [1,2,3,4,5,6]. Click chemistry can be broadly defined as a ligation reaction in which two reactants are joined under ambient conditions to provide the desired product in high chemical yield and short time [7,8,9,10]. Over the last two decades, tremendous development and progress has been achieved in these conjugation reactions to encompass wide substrate scopes in the click reaction. Additionally, in several cases, these ligations proceed in aqueous media without significant decrease of the selectivity and reaction rate. Furthermore, click chemistries enable the facile isolation of the desired products from the reaction mixtures and facilitate the removal of the non-reacted substrates and byproducts, without the need for sophisticated separation methods [11,12,13,14,15,16]. Therefore, click chemistry-based conjugation methods have been applied to several avenues of research, including biochemical sciences, material sciences [17,18,19,20,21,22,23,24], drug discovery [25,26,27,28], pharmaceutical sciences [29,30,31,32,33,34], and synthesis of radiolabeled products [35,36,37,38,39,40,41]. Several typically used ligation reactions which are closely related to click chemistry include the thiol-Michael addition reaction [42], ring-opening reactions of aziridinium ions and epoxides [43], hydrazone and oxime formation from an aldehyde group [44] and so on. However, these reactions showed certain disadvantages such as poor specificity and stability under aqueous conditions, because of the reactivity of these functional groups with biomolecule residues and water. In 2003, K. B. Sharpless and M. G. Finn et al. reported that copper(I)-catalyzed azide-alkyne [3+2] cycloaddition reaction (CuAAC) can be employed as a new class of click reactions for rapid and reliable bioconjugation [45]. As both azide and alkyne groups are unreactive toward protein residues or other biomolecules, this ligation brought about a great impact and has been utilized as an efficient site-specific ligation methodology. Later, some researchers reported that the exogenous metals used to catalyze the click reaction (e.g., copper) could cause mild to severe cytotoxic effects and thus the use of metal catalyst-free chemical reaction has been recommended for several applications [46]. Therefore, catalyst-free, rapid, biocompatible, and bioorthogonal reactions such as strain-promoted azide-alkyne cycloaddition reaction (SPAAC) [47] and inverse-electron-demand Diels–Alder reaction (IEDDA) [48] have been developed as useful alternatives, and have been extensively used in various research fields (Figure 1).

In recent years, these conjugation reactions have also been applied to the synthesis of radioisotope-labeled molecules, which have been used for nuclear imaging using positron emission tomography (PET) and single-photon emission computed tomography (SPECT) as well as for therapeutic applications. Particularly, several important diagnostic radioisotopes including ^11^C (*t*_1/2_ = 20 min), ^18^F (*t*_1/2_ = 110 min), ^99m^Tc (*t*_1/2_ = 360 min), and ^68^Ga (*t*_1/2_ = 68 min) have short half-lives, and thus their radiolabeling procedures require rapid and efficient reactions which can provide reliable radiochemical results, such as high radiochemical yield (RCY) and purity, and minimal undesired by-product formation [49]. In this regard, the catalyst-free click reactions can be highly useful tools for radiolabeling complex small molecules and biomacromolecules, which are sensitive to harsh reaction conditions such as elevated temperatures, extreme pH, and the presence of metal catalysts [50]. In addition to in vitro radiolabeling applications, these ligation methods have also been investigated for in vivo pre-targeted strategies for specific imaging and cancer therapy in animal xenograft models [51].

This review aims to highlight the recent and noteworthy results for the synthesis of radiolabeled molecules using site-specific click reactions. In detail, this review will mainly focus on the following bioconjugation reactions: (1) strain-promoted azide-alkyne cycloaddition (SPAAC); (2) inverse-electron-demand Diels–Alder cycloaddition reaction (IEDDA); (3) rapid condensation/cycloaddition reactions based on electrophilic heterocycles. The review will also showcase the advantages of these reactions, which have empowered radiochemists in the production of radiolabeled products and radiopharmaceuticals for imaging and therapeutic purposes. Finally, future directions and emerging trends of these ligation methods will be discussed.

## 2. Strained Promoted Copper-Free Click Reaction for Synthesis of Radiolabeled Molecules

### In Vitro Radiolabeling of Biomolecules 

**Figure 2:**
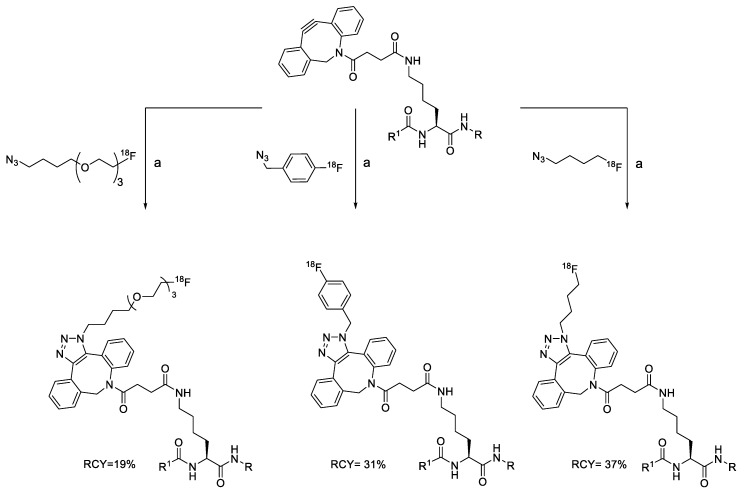
^18^F-radiolabeling of bombesin derivative using SPAAC: **a**) human plasma or dimethyl sulfoxide (DMSO), room temperature, 15 min. R = Pyr-Gln, Pyr = pyroglutamic acid, R_1_ = Leu-Gly-Asn-Gln-Trp-Ala-Val-Gly-His-Leu-Met-NH_2_, RCY = radiochemical yield.

In SPAAC, the ring strain of cyclic alkynes such as dibenzocyclooctyne (DBCO) is used to drive the reaction with azide groups in the absence of copper(I) catalysis [52,53]. Generally, two strategies have been employed for SPAAC-based radiolabeling. The first is the synthesis of radiolabeled cyclooctyne precursors, which can be used for the labeling of azide containing biomolecules, and the other is the preparation of radioisotope-tagged azide tracers which are reacted with cyclooctyne modified biomolecules. In 2011, Feringa group investigated SPAAC reaction for the efficient ^18^F-labeling of biomolecules [54]. In this study, three ^18^F-labeled azides were synthesized, and the prepared tracers were conjugated with DBCO modified bombesin peptide derivatives. Notably, the reaction proceeded with high efficiency to provide ^18^F-labeled cancer-targeting peptides in 15 min with good radiochemical yields (RCYs) (Figure 2). Particularly, radiolabeling studies using these reactions were also explored in human plasma to determine their reactivity and specificity in biological media.

Wuest et al. reported the synthesis of an ^18^F-labeled DBCO analog for efficient preparation of a diagnostic probe. (Table 1, entry 1) This tracer was reacted with several azide group-bearing geldanamycine moieties and carbohydrates to furnish the corresponding ^18^F-labeled products. Importantly, these radiolabeling reactions were performed in various media, including in methanol, DMSO/water (1:1), and bovine serum albumin, wherein the observed RCYs did not decrease significantly [55]. Along similar lines, Carpenter et al. used a modified ^18^F-labeled DBCO analog for the radiolabeling of azide conjugated substrates (Table 1, entry 2). The radiolabeling was performed at room temperature in *N*,*N*-dimethylformamide (DMF) to afford the desired radiolabeled products [56]. The peptide A20FMDV2 (Table 1, entry 3), which has a strong binding affinity with integrin α_v_β_6_-receptor, was successfully labeled with ^18^F, using a SPAAC-based ligation. The radiolabeling of the azide group bearing A20FMDV2 was performed at ambient temperature to give the product in 11% of isolated RCY. The radiolabeled peptide was highly stable in rat serum, and its binding affinity towards the target receptor was not affected. However, in vivo studies revealed its decreased targeting ability due to the structural differences and increased lipophilicity compared to the parent structure [57]. Several other ^18^F-DBCO analogs have shown good RCY for the preparation of radiolabeled peptides for targeting cancer [58,59]. To improve the efficiency of ^18^F radiolabeling, Roche et al. explored a new ^18^F-labeled azide prosthetic group, ^18^F-FPyZIDE (Table 1, entry 6). In their study, the radiolabeled tracers were evaluated in both CuAAC- and SPAAC-based ligations and the labeling results showed that both radiolabeling methods provided high RCYs under mild reaction conditions (room temperature or 40 °C) [60]. Evans et al. investigated the radiosynthesis of ^68^Ga-labeled peptide using azide group-bearing 1,4,7,10-tetraazacyclododecane-tetraacetic acid (DOTA) chelator and DBCO group conjugated cRGD peptide (Table 1, entry 7) [61]. The developed novel bioorthogonal click reaction has been used in the design and preparation of multimodal imaging tracers. Ghosh et al. studied a dual-modal scaffold in which the precursor was first labeled with ^68^Ga using a DOTA chelator, and then, a near-infrared (NIR)-absorbing fluorescent dye, IR Dye 800CW, was incorporated into the tracer using SPAAC ligation. The dual-labeled tracer was then applied to the targeted imaging of a somatostatin receptor and the quantification of its biological uptake in vivo (Table 1, entry 8) [62].

**Figure 3:**
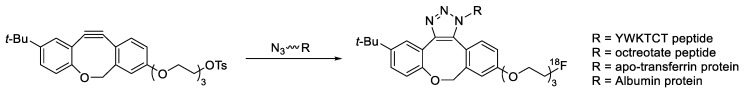
Radiolabeling of peptides or proteins using ^18^F-labeled ODIBO.

Generally, most SPAAC ligations based on DBCO derivatives display second-order rate constants in the 1–2 M^−1^ s^−1^ range with azide groups [63] due to which, the observed RCY is not satisfactory when using low substrate concentrations. To improve reaction kinetics, ^18^F-labeled oxa-dibenzocyclooctyne (ODIBO), which has a k_2_ value of 45 M^−1^ s^−1^, was synthesized to label azide containing biomolecules with high efficiency (Figure 3) [64,65]. This new prosthetic group enabled site-specific radiolabeling using much smaller amounts (about one-tenth) of azide bearing molecules than those of DBCO-based reactions. 

**Figure 4:**
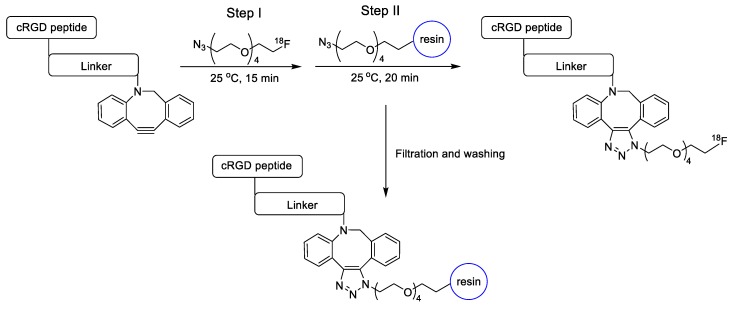
^18^F-radiolabeling of DBCO-modified cRGD dimer using ^18^F-labeled azide precursor and polystyrene-supported azide-modified resin for purification of unreacted substrate.

The study reported by Kim et al. used SPAAC ligation in both radiolabeling reaction and purification steps [66]. For this application, ^18^F-labeled azide tracer was first reacted with DBCO- modified cancer-targeting peptide (cRGD) and then the desired product was separated from unreacted peptide substrates using an azide modified resin as a scavenger for the DBCO group. The remarkable two-steps process provided the radiolabeled peptide in high decay-corrected RCY (92%) and radiochemical purity (98%) (Figure 4). Notably, PET imaging and biodistribution data confirmed the high tumor uptake value of the ^18^F-labeled peptides in U87MG xenograft along with significant enhancement of the tumor to background ratio [67].

**Figure 5:**
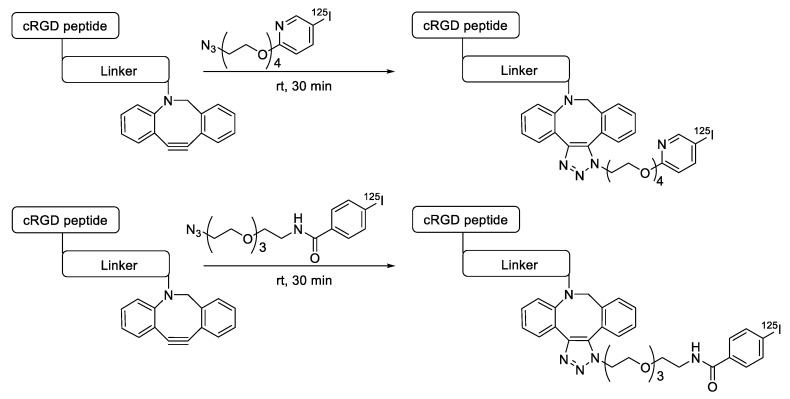
Radiolabeling of DBCO-conjugated cRGD peptide using ^125^I-labeled azide tracers.

**Figure 6:**
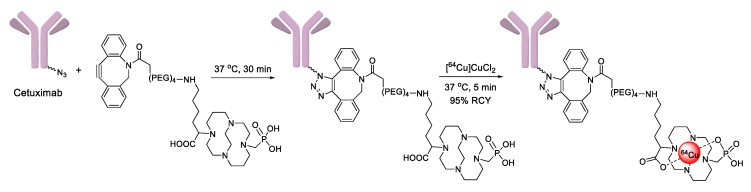
Reaction of azide-conjugated Cetuximab antibody with DBCO-conjugated crossed bridged macrocyclic CB-TE1K1P chelator for ^64^Cu radiolabeling.

SPAAC reaction has also been applied to the labeling of radioisotopes with longer half-lives, such as radioactive metals and radioactive iodine. We reported the use of ^125^I-labeled azide prosthetic groups for synthesizing radiolabeled biomolecules and nanomaterials. In this process, DBCO group modified cRGD peptides were efficiently conjugated with ^125^I-labeled azides in high RCY and radiochemical purity after HPLC purification (Figure 5) [68,69]. It was reported that ^64^Cu could be labeled with cross-bridged cyclam chelator CB-TE1K1P under mild conditions. To employ this chelator for radiolabeling biomolecules, Anderson et al. synthesized a DBCO modified chelator (DBCO-PEG_4_-CB-TE1K1P) and reacted it with an azide-bearing Cetuximab by SPAAC ligation (Figure 6). The ^64^Cu labeling proceeded in high RCY (>95%) at 37 °C, and the radiolabeled antibody showed enhanced serum stability when compared with those of previously reported ^64^Cu chelators [70]. 

**Figure 7:**
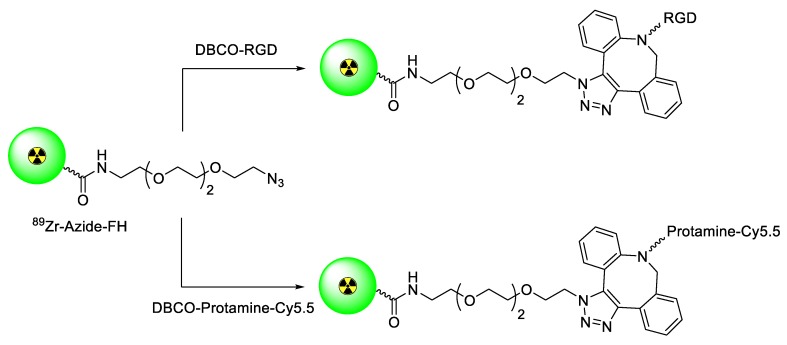
Preparation of ^89^Zr-labeled multifunctional nanoprobes using SPAAC ligation.

Yuan et al. explored the synthesis of ^89^Zr-labeled PET imaging agents using SPAAC ligation on the surface of the superparamagnetic feraheme (FH). For this study, azide-functionalized FH nanoparticles were prepared and were mixed with ^89^Zr under elevated temperature to deliver the ^89^Zr-labeled azide-FH. In the next step, DBCO-conjugated RGD peptide, or Cy5.5 tagged protamine was reacted with ^89^Zr-azide-FH to give the desired radiolabeled products with good radiochemical results and specific radioactivity (Figure 7) [71]. This strategy provided an efficient approach for the preparation of multimodal/multifunctional nanoprobes, which are suitable for a wide range of diagnostic and therapeutic applications. 

**Figure 8:**
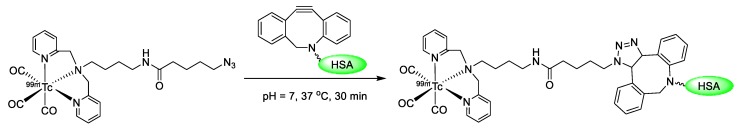
SPAAC for ^99m^Tc-based radiolabeling of human serum protein.

Several kinds of liposomes are known to be useful vehicles for targeted delivery in biomedical research as well as clinically approved platforms [72]. Hood and co-workers used SPAAC ligation for efficient conjugation between ^111^In-labeled liposomes and single-chain variable fragments (scFv) or monoclonal antibodies. The radiolabeled tracer, ^111^In-liposomes-mAb/scFv, was used in the targeted imaging of the platelet-endothelial cell adhesion molecule (PECAM-1) and intracellular adhesion molecule (ICAM-1). The uptake value of ^111^In-liposomes/scFv into the target cells was much higher than that of ^111^In-liposomes/mAb [73]. Recently, thermosensitive hydrogels comprising polyisocyanopeptide (PIC) were labeled with ^111^In via a SPAAC method. In this research, azide-modified PIC hydrogel was first conjugated with DBCO-modified diethylenetriaminepentaacetic acid (DTPA) chelator to afford the PIC-DTPA conjugate. Next, PIC-DTPA was reacted with ^111^InCl_3_ to give the ^111^In-labeled PIC in high RCY. The radiolabeled PIC was applied in a SPECT imaging study for evaluating the efficacy of PIC gels in wound mouse models [74]. Figure 8 shows the ^99m^Tc labeling of human serum albumin (HSA) via a SPAAC reaction. After labeling ^99m^Tc(CO)_3_ with an azide group-modified dipyridine chelator, it was then reacted with ADIBO bearing HSA under mild condition to give the radiolabeled protein in high RCY (76–99%). The ^99m^Tc-labeled HSA prepared by this procedure showed better stability in vivo, as compared with those previously reported ^99m^Tc-labeled HSA, which were obtained by direct ^99m^Tc labeling [75]. The radiolabeled HSA thus prepared, was used in blood pool imaging using SPECT.

## 3. Inverse-Electron-Demand Diels–Alder Reaction for Synthesis of Radiolabeled Molecules

### 3.1. In Vitro Radiolabeling of Biomolecules 

**Figure 9:**
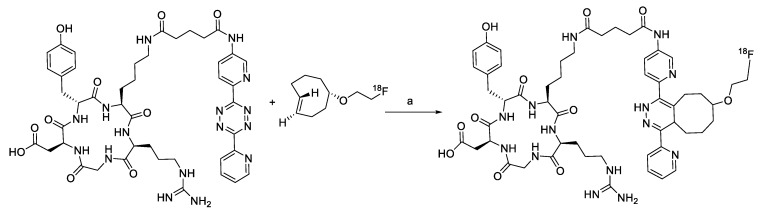
Radiolabeling of tetrazine conjugated cRGD peptide using ^18^F-labeled TCO; **a**) DMSO, 10 s, room temperature.

The inverse electron demand Diels–Alder (IEDDA) between 1,2,4,5-tetrazine and strained alkene (such as trans-cyclooctene, TCO) is a well-established bioorthogonal reaction, which is typically regarded as the fastest click reaction with first-order rate constants ranging up to 10^5^ M^−1^ S^−1^ [76,77,78,79] Since the first report on IEDDA reaction, several kinds of strained alkenes/alkynes and tetrazine analogs have been synthesized, and these functional group pairs have been applied to the radiolabeling of various small molecules, biomolecules, and nanomaterials [80,81]. Due to the extremely rapid reaction rate of IEDDA under mild conditions such as room temperature, neutral pH, and in aqueous media, this reaction has been a highly useful ligation approach for labeling radioisotopes with short half-lives. In 2010, Fox et al. reported the IEDDA-mediated ^18^F-labeling of small molecules. The radiolabeled TCO (Table 2, entry 1) could be synthesized by a nucleophilic substitution reaction of the tosylated precursor in 71% RCY. Remarkably, the IEDDA reaction between a model tetrazine substrate and ^18^F-labeled TCO provided the desired product in more than 98% RCY in 10 seconds [82]. Conti et al. applied IEEDA to the synthesis of an ^18^F-labeled cancer-targeting peptide [83]. The labeling reaction of a tetrazine conjugated cRGD peptide was carried out using an ^18^F-labeled TCO analog, which was prepared using a similar protocol, and delivered the radiolabeled peptide in excellent RCY (Figure 9). The ^18^F-labeled cRGD thus prepared, was evaluated in the U87MG xenograft model and exhibited clear visualization of tumor cells by PET imaging.

Later, the same group reported a maleimide-conjugated tetrazine analog, which was used to incorporate the tetrazine group onto biomolecules comprising free cysteine moieties. The tetrazine bearing biomolecules (cRGD peptide and VEGF protein) prepared by the above method was then reacted with ^18^F-labeled TCO to give PET-imaging tracers for diagnosis of cancer cells in vivo (Table 2, entry 2) [84]. Weissleder and coworkers synthesized ^18^F-AZD2281, a poly-ADP-ribose-polymerase 1, as a PET imaging tracer (Table 2, entry 3). In this report, ^18^F-labeled TCO and a tetrazine group-bearing AZD2281 were incubated for 3 minutes, and the crude product was purified using a magnetic TCO-scavenger resin for removing the unreacted substrate, without the need for carrying out the traditional HPLC purification. The process delivered the ^18^F-labeled AZD2281 in 92% RCY using the scavenger-assisted method [85]. The prepared radiolabeled tracer was then evaluated in xenograft models to visualize MDA-MB-436 tumors. Wu and coworkers extended the application of IEDDA ligation to the radiolabeling of the exendin-4 peptide and applied the ^18^F-labeled exendin-4 to the targeted imaging of GLP-1R receptor in an animal model [86]. In 2015, the Schirrmacher group reported the novel silicon-fluoride acceptor (SiFA) labeling method, which is based on an isotopic exchange reaction (Table 2, entry 5). This simple labeling step (^19^F →^18^F), which is based on a silicon-fluorine scaffold, provided the ^18^F-labeled tetrazine in much higher RCY (78%) than those realized with other ^18^F chemistries [87].

Norbornene analogs are known to be reactive toward tetrazines. Although the reaction rate was much slower than those of TCO analogs, the preparation of a norbornene substrate is straightforward. Importantly, norbornene analogs are known to be more stable than TCO analogs, which are prone to isomerization to their *cis*-isomers under physiological conditions. Knight and coworkers reported the reaction of the tetrazine group-conjugated bombesin peptide with an ^18^F-labeled norbornene prosthetic group, to provide the radiolabeled product with high efficiency and radiochemical purity (Table 2, entry 6) [88]. In addition to the radioactive fluoride, ^11^C is another important cyclotron-produced radioisotope for preclinical and clinical PET imaging. Particularly, the ^11^C-labeled methyl triflate and methyl iodide are the most prominent synthons for nucleophilic methylation of alcohols, amines, and thiols, which are commonly used for the production of various radiotracers and radiopharmaceuticals. Herth and coworkers reported the first synthesis of an ^11^C-labeled tetrazine and its reaction with a strained cyclooctene (Table 2, entry 7) [89]. The radioactive precursor [^11^C]CH_3_I was reacted with a tetrazine-conjugated phenol group to give the desired radiolabeled tetrazine in 33% RCY, which underwent a click reaction with a trans-cyclooctenol in 20 seconds, suggesting the suitability of this conjugation method for preparation of radiolabeled molecules with short-lived isotopes such as ^11^C. Devaraj et al. reported the development of a ^68^Ga-labeled tetrazine modified dextran polymer for increasing the half-life and in vivo stability of the tracer in blood (Table 2, entry 8), and evaluated its use in human colon cancer cells (LS174T) and xenograft models [90].

Radioactive iodines have been used for the preparation of various radiotracers for in vivo imaging and biodistribution studies. The traditional radioiodination method via an electrophilic substitution reaction typically provides high RCY in a short time. However, the radiolabeled tracer synthesized using the above reaction generally exhibited considerable deiodination in the living subjects, and the liberated radioactive iodines rapidly accumulated in the thyroid and stomach which and resulted in high background signals in the images. Moreover, the use of a strong oxidant that requires radioiodination often resulted in decreased biological activity of the molecules. To address these problems, the radioactive iodine-labeled tetrazine can be used as an alternative method for the efficient radiolabeling of biomolecules. Valliant et al. reported the rapid radiolabeling of antibody based on IEDDA. In this study, the ^125^I-labeled tetrazine analog was incubated with the TCO-modified anti-VEGFR2 for 5 minutes to afford the desired product in 69% RCY. Interestingly, the radiolabeled antibody, which was prepared using this procedure, displayed a 10-fold increase in stability to in vivo deiodination, then the same antibody prepared by direct radioiodination using iodogen (Table 2, entry 9) [91]. Along similar lines, we investigated a modified ^125^I-labeled tetrazine tracer via oxidative halo-destannylation of the corresponding precursor (Table 2, entry 10) [92]. The prepared radiolabeled tetrazine was then applied to the labeling of TCO derived cRGD peptide and human serum albumin (HSA) and delivered excellent RCYs (>99%). The biodistribution study of the ^125^I-labeled HSA in normal ICR mice demonstrated enhanced in vivo stability toward deiodination than the radiolabeled HSA obtained using the conventional iodination method. Valliant group also synthesized ^123/125^I-labeled carborane-tetrazine and employed it for the radiolabeling of TCO-bound H520 cells [93].

**Figure 10:**
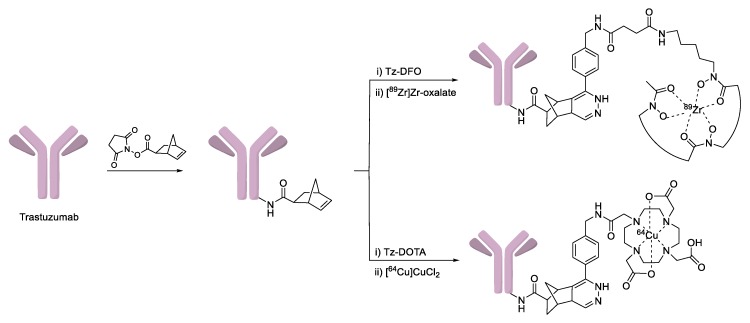
IEDDA-mediated radiolabeling of trastuzumab.

**Figure 11:**
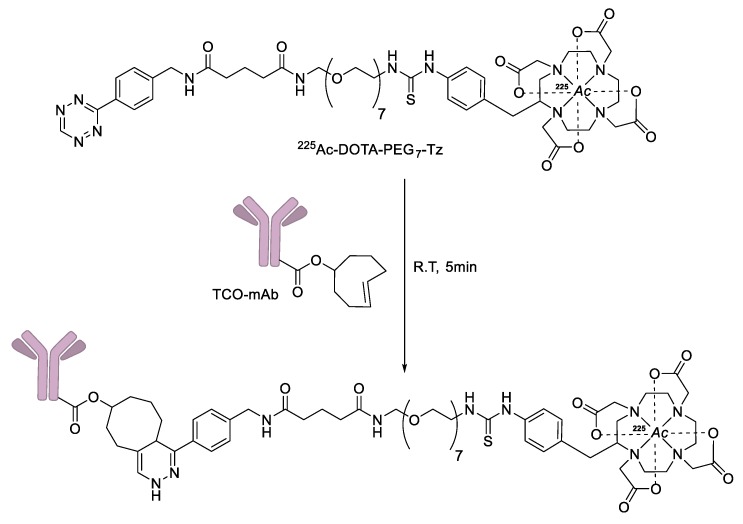
IEDDA-mediated synthesis of ^225^Ac-labeled monoclonal antibody.

Several radioactive metal-labeled tracers have also been prepared by IEDDA ligation for diagnostic purposes. Lewis et al. reported tetrazine conjugated metal-chelating agents such as DOTA and deferoxamine (DFO) for the radiolabeling of norbornene bearing trastuzumab using ^64^Cu or ^89^Zr (Figure 10) [94]. By using this procedure, radiolabeled trastuzumab was obtained in high RCY (>80%) and high specific radioactivity (>2.9 mCi/mg). Furthermore, PET imaging studies demonstrated that radiolabeled antibodies were quite stable in vivo conditions and showed specific uptake in HER2-positive BT-474 tumor cells. In 2018, IEDDA ligation was employed for the preparation of therapeutic radioisotope-labeled human antibodies 5B1 and huA33 (Figure 11) [95]. In this study, a tetrazine conjugated DOTA chelator was synthesized, and labeled with ^225^Ac, a useful therapeutic radioisotope. The radiolabeled tetrazine tracer was then reacted with TCO-modified antibodies to give the desired products within 5 min. This two-step method provided superior RCYs compared to the conventional approaches used in clinical applications. In addition, the biodistribution results demonstrated that the ^225^Ac-labeled antibody showed high tumor uptake values and relatively low non-specific accumulation in normal organs.

**Figure 12:**
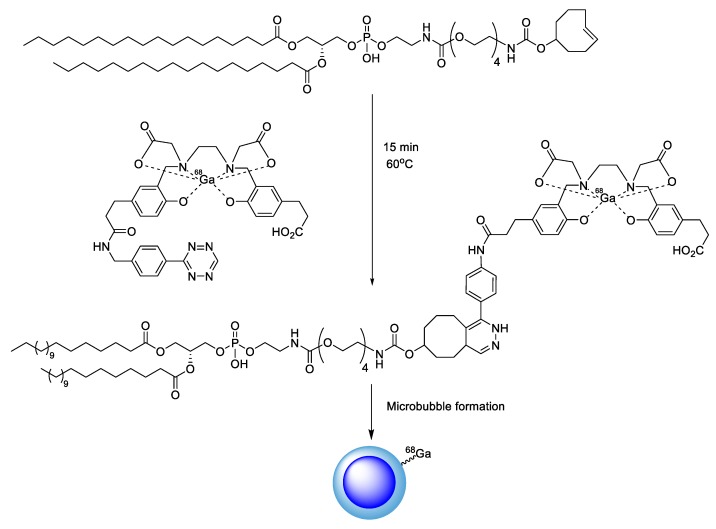
Synthesis of ^68^Ga-labeled microbubble using IEDDA.

Recently, Long et al. reported the radiolabeling process for microbubble, which is a contrast agent used in ultrasound imaging and relies on an IEDDA reaction for its operation. First, a tetrazine-bearing metal chelator (HBED-CC) was labeled with ^68^Ga. The TCO-modified phospholipids were then treated with ^68^Ga-HBED-CC-tetrazine under mild conditions to give the ^68^Ga-labeled lipid molecule (^68^Ga-PE). Next, the prepared ^68^Ga-PE was combined with other types of lipids, and the resulting formulation was activated to form gas-filled microbubbles (Figure 12). This strategy enabled the PET-based real-time monitoring and pharmacokinetic study of newly developed contrast agents for ultrasound analysis [96].

### 3.2. In Vivo Pre-Targeted Imaging and Therapy

**Figure 13:**
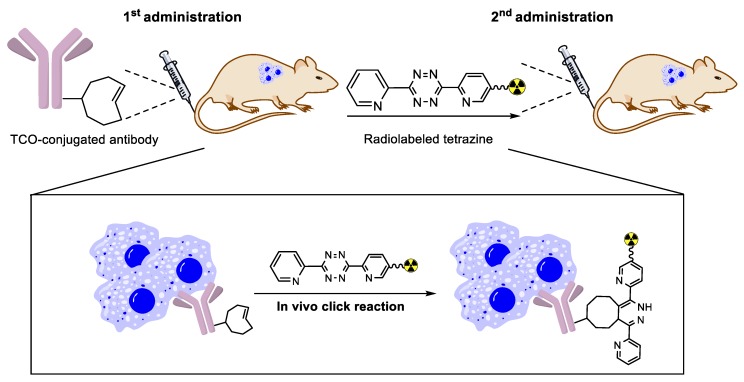
General strategy for pre-targeted imaging and therapy using IEDDA.

The tetrazine and TCO groups are not reactive towards amine or thiol nucleophiles and show high reaction specificity in biological media. In addition, IEDDA can proceed with fast kinetics even at very low reactant concentrations. Due to these reasons, IEDDA-based ligation is one of the most potent tools for pre-targeting applications among the existing click reaction approaches. In the pre-targeted approach, a cancer-targeting ligand and a radiolabeled small molecule are administered separately into a living subject. Generally, the TCO (or tetrazine) conjugated tumor-targeting antibody is injected first into the tumor xenograft model and is allowed to accumulate in the tumor cells for a certain period (Figure 13). Next, the radiolabeled tetrazine (or TCO) group is administered after the excess amount of antibody in healthy tissues is excreted from the body. The in vivo click reaction through the above procedure decreases the circulation time of the radioligand and results in reduced non-specific uptake of radioactivity in healthy tissues. Furthermore, this approach also facilitates the delivery of radioisotopes with short half-lives, which would not be feasible with antibody-based imaging studies [97]. Table 3 shows in vivo pre-targeted studies that were conducted using IEDDA-based ligation in animal models.

**Figure 14:**
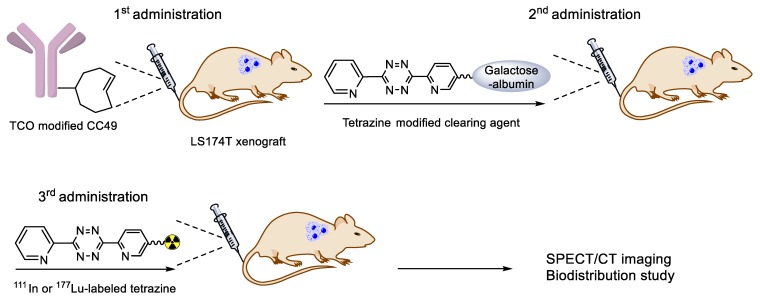
A modified strategy using tetrazine-bearing clearing agent.

In 2010, the Robillard group reported their pioneering work on in vivo pre-targeted imaging of cancers using IEDDA ligation [98]. In the first step, TCO group bearing CC49 antibody was injected to target colon cancer cells in a mouse model. Post administration of the antibody (24 h), only a small excess (3.4 equivalent) amount of ^111^In-labeled tetrazine tracer was injected into the same mouse model. The obtained SPECT images showed the efficient delivery of the radioisotope into the tumor and indicated a high tumor-to-normal tissue ratio (Table 3, entry 1). In the next study, the same research group revealed that TCO could be converted to its (*Z*)-isomer, which is unreactive to tetrazine in the presence of copper-containing proteins [99]. Thus, a shorter linker was introduced in the tetrazine tracer to impede interactions with the copper-containing proteins in albumin. By this structural modification, the reactivity and isomerization half-life of TCO was increased compared to that of the previously used TCO analog. Later, Robillard and coworkers reported the use of tetrazine-functionalized clearing agents as a modified pre-targeting system (Table 3, entry 3) [100]. While a portion of the administered antibody accumulated in the tumor tissue in this approach, a significant portion of it still remained in the blood. This accumulated antibody could cause a reduced target-to-background ratio because IEDDA reaction is also feasible at non-specific areas in the body. To address this problem, the group added one more step in the animal study (Figure 14). After the TCO-modified antibody was injected into the xenograft model to target tumor cells in vivo, the tetrazine bearing galactose-albumin conjugate was injected as a TCO clearance agent to mask the unbound TCO-modified antibody in the blood. The radiolabeled tetrazine was then injected to enable the IEDDA reaction at the surface of tumor site. This approach demonstrated that the use of a clearing agent could lead to the doubling of the tetrazine tumor uptake and a greater than 100-fold improvement of the tumor-to-blood ratio at 3 h could be realized after injection of the radiolabeled tetrazine.

The same group reported a pre-targeted radioimmunotherapy study using a similar strategy. To achieve high tumor uptake and improved tumor-to-blood ratio, the group employed a linker with higher hydrophilicity to prepare the TCO-tagged CC49 antibody [101]. In 2015, TCO-functionalized diabody, AVP04-07 was evaluated in the pre-targeted strategy [102]. In this study, the TAG72-targeting dimers of single-chain Fv fragments and ^177^Lu-labeled tetrazine tracers were evaluated in the LS174T tumor xenograft. As the diabody showed rapid renal clearance kinetics, this strategy could provide high tumor-to-blood ratio and low non-specific retention in the kidneys. In a related study, the authors successfully performed an IEDDA-based pre-targeted study by employing HER2 affibody molecules and ^111^In/^177^Lu-labeled tetrazine tracers (Table 3, entry 6) [103]. 

**Figure 15:**
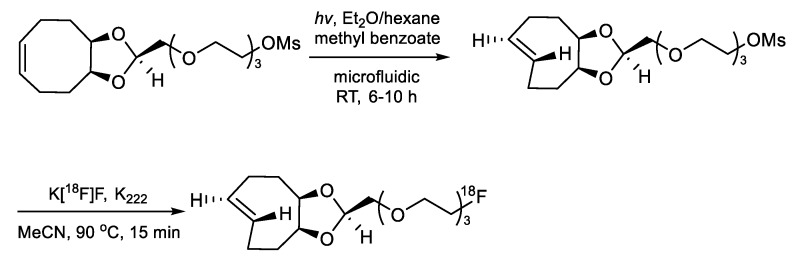
Radiosynthesis of ^18^F-labeled TCO.

**Figure 16:**
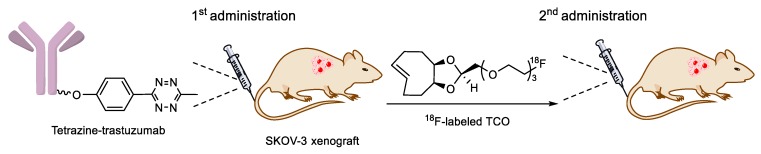
Two-step pre-targeting strategy using ^18^F-labeled TCO.

In addition to these results, several research groups have investigated a variety of pre-targeted approaches using short half-life radioisotope-labeled TCO or tetrazine derivatives. Denk et al. developed a novel ^18^F-labeled tetrazine by the direct ^18^F-fluorination of the tosylated precursor, which proceeded in an RCY up to 18% (Table 3, entry 7) [104]. The PET imaging study exhibited fast homogeneous biodistribution of the ^18^F-labeled tetrazine, which can also cross the blood–brain barrier. The high reactivity of this tracer towards TCO-bearing molecules and favorable pharmacokinetic properties indicated that ^18^F-labeled tetrazine can be a useful tracer for bioorthogonal PET imaging. Lewis et al., reported ^18^F-based pre-targeted PET imaging studies using TCO-modified anti-CA19.9 antibody 5B1 and a 1,4,7-triazacyclononane-1,4,7-triacetic acid (NOTA)-conjugated tetrazine analog. The complexation reaction using AlCl_3_ and [^18^F]F^−^ provided the desired radioligand in 54–65% decay-corrected RCY [105]. The in vivo pre-targeted images displayed its effective targeting ability with radioactivity up to 6.4% ID/g in the tumors at 4 h post administration. Sarparanta and co-workers investigated in vivo IEDDA reaction between TCO conjugated monoclonal antibodies and ^18^F-labeled tetrazine molecule [106]. For this study, TCO conjugated antibody (trastuzumab and cetuximab) was injected into tumor-bearing (BT-474 cells and A431 cells) mice and the ^18^F-labeled tetrazine-containing hydrophilic linker was injected into the same xenograft models after given time points (1, 2, or 3 days). The highest tumor-to-background ratio was observed when the radioisotope was injected after 3 days post the administration of the TCO-modified antibody. In addition, the ^18^F-labeled tetrazine was applied to the pre-targeted in vivo imaging of TCO-modified porous silicon nanoparticles (Table 3, entry 10) [107]. Bormans et al. developed a new ^18^F-labeled TCO tracer for in vivo IEDDA. To prepare radiolabeled TCO, the authors synthesized a dioxolane-fused TCO analog from its cis isomer by using a micro-flow photochemistry process (Figure 15) [108]. The nucleophilic substitution of mesylated precursor using dry K[^18^F]F, K_222_ complex provided the desired ^18^F-labeled tracer in 12% RCY and >99% radiochemical purity. This product showed excellent reactivity and stability toward a tetrazine and thus it was applied to pre-targeted PET Imaging. In this approach, a tetrazine-modified trastuzumab monoclonal antibody was injected initially into SKOV-3 xenograft models (Figure 16). After 2 or 3 days, the ^18^F-labeled TCO was injected, and then the PET images were obtained after 2 h post the administration of the radioligand. The obtained results showed that the pre-targeted imaging strategy provided better tumor-to-muscle ratio when compared to that of control groups which did not use the pre-targeting approach (Table 3, entry 12) [109].

**Figure 17:**
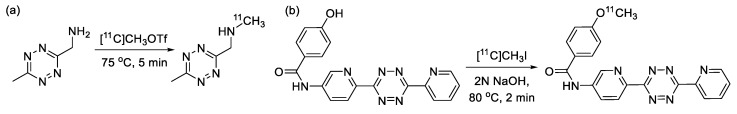
^11^C-labeled tetrazine tracers for in vivo IEDDA reaction, (**a**) from ref [111], (**b**) from ref [112].

The radiolabeled small molecule tracers often underwent rapid renal or hepatobiliary clearance, and therefore, the efficiency of in vivo click reactions is reduced. To increase the blood circulation time of the functional group, the Weissleder group designed the tetrazine group-bearing polymers comprising dextran scaffolds [110]. An ^18^F-labeled polymer-modified tetrazine and TCO-bearing CD45 monoclonal antibodies were investigated in a living mouse, and the PET imaging study revealed excellent conversion of reactants and high tumor uptake in the tumor xenograft, which suggested that the radiolabeled polymer will be a promising candidate for pre-targeted imaging. The use of IEDDA for pre-targeted PET imaging has also been investigated with ^11^C. In 2016, Mikula et al. reported the use of ^11^C-labeled tetrazine for in vivo click reaction. An amino tetrazine analog was reacted with [^11^C]CH_3_OTf to provide ^11^C-labeled tetrazine in 52% of RCY (Figure 17) [111], and the resulting product exhibited high reaction rate with TCO derivatives. Furthermore, the product also showed good stability under physiological conditions and demonstrated rapid clearance kinetics in mice. This ^11^C-labeled tracer was then applied to animal imaging studies with TCO-modified mesoporous silica nanoparticles in normal mice. Herth et al. reported the improved radiosynthesis of ^11^C-labeled tetrazine for pre-targeted PET imaging (Table 3, entry 15) [112]. In this study, the radioligand was evaluated with TCO-functionalized polyglutamic acid and indicated potential use for brain imaging. 

The use of the TCO-tetrazine ligation in living subjects was also extended to several metal radioisotopes. Lewis et al. reported a pre-targeted strategy using a TCO-bearing huA33 antibody and ^64^Cu-labeled NOTA-tetrazine conjugate (Table 3, entry 16). In this study, a tumor-targeted antibody was administered to SW1222 colorectal cancer xenografts, and the tetrazine tracer was then injected one-day post administration of the antibody. This approach exhibited enhanced tumor-to-background ratio and reduced non-specific radiation dose in normal tissues [113]. In the following study, the authors reported a site-specific conjugation method to construct the huA33-TCO immunoconjugate by using enzymatic transformations and a bifunctional linker [114]. A similar bioconjugation strategy was also applied to the preparation of a TCO and fluorescent dye-bearing antibody (huA33-Dye800-TCO) for bimodal PET/optical pre-targeted imaging of colorectal cancer cells (Table 3, entry 19) [116] using a ^64^Cu-sarcophagine-based tetrazine tracer. This strategy demonstrated the non-invasive visualization of tumors and the image-guided excision of malignant tumor tissue. Aboagye et al., synthesized ^68^Ga-labeled tetrazine to study its use in pre-targeted PET imaging of EGFR-expressing A431 tumor. After administration of the TCO-bearing cetuximab, the ^68^Ga-labeled tracer was injected to the mouse model, and PET imaging showed a significant improvement in the tumor-to-background ratio compared to that with the traditional direct radiolabeling method [117]. Recently, Lewis et al. used a modified pre-targeted PET imaging strategy for obtaining a better tumor-to-blood ratio. The authors employed a tetrazine-modified dextran polymer to reduce injected TCO-bearing antibody, which remained in blood circulation. After the TCO-modified antibody was injected into the xenograft model to target tumor cells, the TCO scavenger was administrated to mask unbounded TCO modified antibody in the blood. Next, ^68^Ga-labeled tetrazine radioligand was injected to allow the IEDDA reaction at the surface of tumor cells. Further, the use of the TCO masking agent in this study showed a significant improvement in the PET image quality and tumor-to-background ratio (Table 3, entry 21) [118].

**Figure 18:**
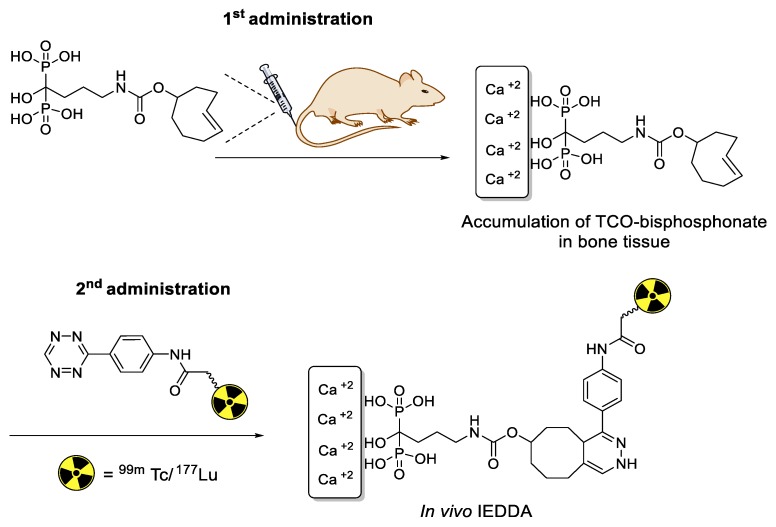
Pre-targeted IEDDA ligation between TCO-bisphosphonate and radiolabeled tetrazine in bone tissue.

The Valliant group demonstrated a pre-targeted strategy for bone imaging and radiotherapy based on the IEDDA between the TCO-conjugated bisphosphonate and radiolabeled tetrazines (Figure 18) [119]. In this experiment, TCO-bisphosphonate conjugate was first injected into an animal model for accumulation of the dienophile in the skeleton. After 12 h post administration, ^99m^Tc-labeled tetrazine was administered intravenously, and the acquired SPECT/CT imaging revealed high radioactivity in the knees and shoulder, which suggested that the TCO-bisphosphonate can be a useful probe for targeting functionalized tetrazine in the bone tissue. A therapeutic radioisotope (^177^Lu)-labeled radioligand was also investigated in the same study.

In 2018, Garcia et al. investigated an antibody pre-targeting approach using TCO-bearing bevacizumab and ^99m^Tc-labeled tetrazine tracer. To increase renal clearance kinetics of the radioisotope, a hydrophilic peptide linker was introduced between tetrazine and the 6-hydrazinonicotinyl group, which is a well-known chelator of ^99m^Tc. The pre-targeted bevacizumab SPECT imaging was then investigated in B16-F10 melanoma cell’s xenograft [120]. In addition to various diagnostic research, the alpha-particle emitting radioisotope (^212^Pb) was applied to the pre-targeted radioimmunotherapy by Quinn et al. (Table 4, entry 24). In this study, the LS174T tumor-bearing mice were injected with CC49-TCO monoclonal antibody. Two doses of the tetrazine bearing Galactose-albumin as a TCO clearing agent were injected after 30 and 48 h to remove the unbound antibodies in blood and normal organs. Then, ^212^Pb-labeled tetrazine was injected for targeted tumor therapy. This pre-targeted alpha-particle therapy successfully reduced the tumor growth and improved the survival of model mice [121].

## 4. Other Click Reactions Based on Aromatic Prosthetic Groups

### 4.1. Condensation/Addition Reactions Using Aromatic Compounds

As shown in previous sections, SPAAC and IEDDA have been two of the most frequently used radiolabeling methods for several years. To apply these reactions in the labeling procedure, the target molecule (e.g., peptide, antibody) needs to be modified to incorporate an artificial functional group, which is then reacted with the radiolabeled prosthetic group. For example, a TCO analog needs to be conjugated with the target molecule, to facilitate its reaction with a radioisotope-containing tetrazine. Such modification of biomolecule requires additional synthetic, and purification steps. Furthermore, the presence of excess amounts of randomly conjugated functional groups can cause decreased biological activities of the molecules. Therefore, several labeling procedures, which do not involve a modification of the biomolecules, have been developed. In many cases, these methods utilized electrophilic aromatic prosthetic groups that displayed rapid reaction rates and high selectivities toward a specific nucleophile such as thiol or 1,2-amino thiol. Table 4 summarizes recent studies on the applications of aromatic prosthetic groups for radiolabeling reactions.

In 2012, Jeon et al. investigated the rapid condensation reaction between ^18^F-labeled 2-cyanobenzothiazole (^18^F-CBT) and *N*-terminal cysteine-bearing biomolecules (Table 4, entry 1) [122]. The ^18^F-CBT was synthesized from the corresponding tosylated precursor using K[^18^F]F and 18-crown-6 as the phase transfer catalyst. This radiolabeled CBT (^18^F-CBT) can be reacted with *N*-terminal cysteine with a second-order reaction rate of ca. 9 M^−1^ s^−1^. The rapid condensation reaction between the *N*-terminal cysteine-bearing dimeric cRGD peptide and the ^18^F-CBT provided the ^18^F-labeled peptide (^18^F-CBT-RGD_2_) in a high (>80%) RCY under mild conditions, and the prepared ^18^F-CBT-RGD_2_ was investigated for its use in specific tumor imaging in U87MG xenograft models. Later, ^18^F-CBT was also applied for the efficient radiolabeling of EGFR-targeting affibody molecules (Z_EFGR:1907_), and the radiolabeled affibody provided clear visualization of the A431 tumors in animal models [123]. As the heterocyclic adducts, which result from the condensation reaction between CBT and *N*-terminal cysteine are hydrophobic, the injected tracers prepared by the above method showed high non-specific uptake in normal organs. Therefore, the Seimbille group synthesized a more hydrophilic ^18^F-labeled CBT tracer containing a diethylene glycol linker and 2-fluoropyridine moiety (Table 4, entry 2) The optimized radiolabeling condition provided ^18^F-labeled cancer-targeting peptide, which is more hydrophilic than the ones reported in the previous studies [124]. The same research group also reported the synthesis of the metal-chelating agent-conjugated CBT prosthetic groups for ^68^Ga-labeled tracers for PET imaging of tumor hypoxia [125]. The rapid condensation for radiolabeling procedure provided the desired radiotracers in high RCY under mild conditions (Table 4, entry 3). In another study, the same group synthesized the two bifunctional chelators, the desferrioxamine B-bearing CBT (DFO-CBT) and the cysteine-bearing CBT (DFO-Cys) for efficient radiolabeling. These chelators were employed in the labeling with the [^89^Zr]Zr-oxalate and rapid conjugation with cRGD peptide. The two-step radiochemical process exhibited high RCY under mild reaction conditions [126]. As CBT structure contained a hydroxy group, it can be a good substrate for facile labeling of radioactive iodines [127]. Thus, we synthesized a ^125^I-labeled CBT (^125^I-CBT) via electrophilic iodination reaction under mild reaction conditions. The ^125^I-CBT was then applied to the rapid radiolabeling of *N*-terminal cysteine-bearing cRGD peptide in high RCY (Table 4, entry 4). 

In 2013, Barbas III group reported the chemoselective ligation of thiol-containing proteins using methylsulfonyl derivatives [128]. They showed that phenyloxadiazole methylsulfone and phenyltetrazole methylsulfone react rapidly and selectively with the sulfhydryl group of cysteine residues in aqueous media under mild conditions (at neutral pH and room temperature) In addition, the structures resulting from these ligation reactions were more stable under physiological conditions in comparison to the corresponding products obtained by maleimide-thiol chemistry. These advantages lead to the development of new prosthetic groups for site-specific radiolabeling reactions. Mindt et al. reported a ^18^F-labeled phenyloxadiazole methylsulfone analog([^18^F]FPOS) for the rapid and chemoselective radiolabeling of thiol-bearing biomolecules under mild conditions (Table 4, entry 5) [129]. In this study, [^18^F]FPOS was applied to efficient radiolabeling of free thiol group-bearing biomolecules. The radiolabeled affibody (Z_HER2:2395_) could be successfully applied to the PET imaging of HER2-positive tumor cells in animal models. Recently, we have reported a radioiodinated phenyltetrazole methylsulfone derivative as a new thiol-reactive prosthetic group (Table 4, entry 6) [130]. The ^125^I-labeled (4-(5-methane-sulfonyl-[1,2,3,4]tetrazole-1-yl)-phenol) (^125^I-MSTP) can be prepared by using a simple iodination reaction from the phenolic precursor in high RCY (73% isolated yield) and purity (>99%). The ^125^I-MSTP was used for site-specific radiolabeling of a single free-thiol-bearing peptide and protein by using radioiodinated labeling of thiol-containing biomolecules. The radiolabeled HSA prepared by this method exhibited enhanced in vivo stability upon deiodination compared with radioiodinated HSA prepared by a direct iodination reaction. In 2018, Park et al. reported a novel condensation reaction using an aryl diamine linker and ^125^I-labeled aldehyde prosthetic group (Table 4, entry 7) [131]. This method was applied to rapid and efficient radiolabeling of bioactive molecules and the labeled products showed high in vitro and in vivo stability. Samnick et al. proposed a new phenol-reactive prosthetic group for site-specific radiolabeling reaction of tyrosine-containing biomolecules (Table 4, entry 8) [132]. The ^18^F-labeled 1,2,4-triazoline-3,5-dione([^18^F]FS-PTAD) was reacted with the model compounds such as phenol, l-tyrosine and *N*-acetyl-l-tyrosine methyl amide to evaluate the efficacy of the labeling reaction, which proceeded rapidly under mild aqueous conditions to furnish the corresponding radiolabeled compounds in good RCY (45–58%) within 5 min.

### 4.2. Miscellaneous

**Figure 19:**
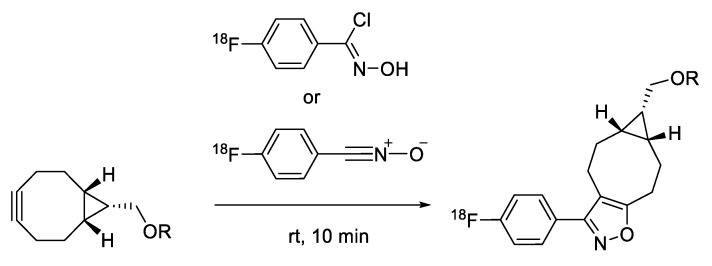
[3+2] cycloaddition reaction using ^18^F-labeled nitriloxides or *N*-hydroxyimidoyl chloride.

Neumaier and coworkers demonstrated the ^18^F-radiolabeling of biomolecules using [3+2] cycloaddition reactions between ^18^F-labeled nitrone and maleimide-bearing molecules [133]. This reaction can provide high efficiency for the synthesis of radiolabeled small molecules. However, the cycloaddition reaction required elevated temperatures in organic solvents, and thus, this method was not suitable for radiolabeling of proteins or antibodies. Continuing this theme, the same group explored more efficient [3+2] cycloaddition reactions using ^18^F-labeled nitriloxides and *N*-hydroxyimidoyl chloride (Figure 19). Interestingly, these radiolabeled tracers showed high reactivity with a strained alkene and norbornene analogs under ambient temperature, suggesting that this method can be a useful alternative to the copper-free azide–alkyne click reactions for the radiolabeling of biomolecules [134].

**Figure 20:**
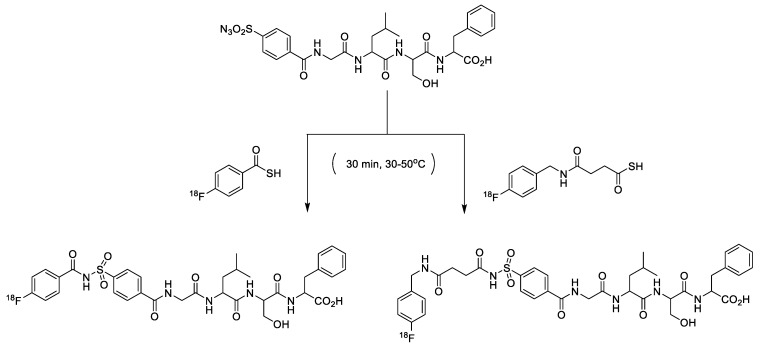
Radiolabeling of biomolecules using sulfo-click chemistry.

Recently, Wuest et al. demonstrated the first application of the sulfo-click chemistry in the ^18^F-labeling reaction (Figure 20). In this study, ^18^F-labeled thiol acids were synthesized and treated with sulfonyl azide-modified small molecules and peptide substrates to afford the corresponding radiolabeled products in moderate to good RCYs [135]. Furthermore, this labeling reaction can be selectively performed in aqueous solvents with a high degree of functional group compatibility.

**Figure 21:**
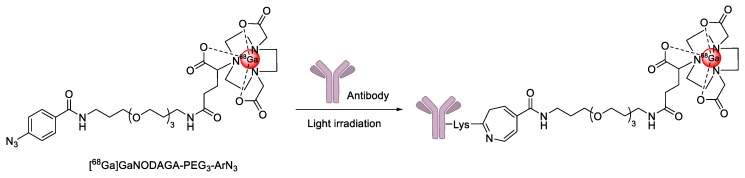
Radiolabeling of antibodies using photochemical conjugation reaction.

Recently, a novel photochemical conjugation reaction was developed for one-pot radiolabeling of antibodies by the Holland group [136,137]. The group synthesized the ^68^Ga-labeled photoactivatable ligand, which contained an aryl azide group ([^68^Ga]GaNODAGA-PEG_3_-ArN_3_) (Figure 21). The prepared radiotracer underwent a facile reaction with an amino group of the antibodies, including GMP-grade Herceptin^TM^ upon light irradiation (λ_max_ ~ 365 nm) within 5 min. The radiolabeled product was also utilized for the specific tumor imaging in SK-OV-3 tumor xenograft. A similar method was also applied to the radiosynthesis of ^89^Zr-labeled antibody by using a desferrioxamine B conjugated aryl azide group [138]. As the radiolabeling of trastuzumab has been carried out over a short time with high efficiency and purity, this approach will be applicable for the efficient radiolabeling of various biologically active molecules.

## 5. Conclusions and Future Perspectives

In this review, we focused on recent examples that highlight the application of bioorthogonal click chemistries for the preparation of radiolabeled products. For many years, rapid and selective conjugation reactions including SPAAC and IEDDA have been successfully employed for the straightforward, site-specific, and efficient labeling of various radioisotopes to the small molecules, biomacromolecules, functional nanomaterials, and living cells. In addition, electrophilic aromatic prosthetic groups which display fast reaction kinetics and high selectivity towards the amine or thiol groups could also be the preferred methods for the radiolabeling procedure, because these reactions do not need the introduction of an artificial functional group to the target the biomolecule. Regarding future perspectives, it is anticipated that the relevance of bioorthogonal strategies will continue to be applicable beyond the rapid labeling of a radioisotope to a target molecule of interest. For example, the development of in vivo ligation based on IEDDA enabled the investigation of various approaches for specific tumor imaging with decreased non-specific accumulation of radioligand in normal tissues. Particularly, the introduction of clearing agents before administration of radiotracers demonstrated improved tumor-to-background ratio with enhanced uptake values in the target sites. Although some recent advancements can provide potent tools in nuclear medicine, several key challenges need to be addressed for their further development. The functional groups and resulting adducts obtained by bioorthogonal ligations are normally hydrophobic, which may result in non-specific uptake and retarded excretion kinetics in a living subject. Moreover, conjugation of a relatively large functional group to the small molecule probes or short peptides will affect their pharmacokinetic profiles and induce undesired accumulation or retention of radioactive signals in healthy tissues. For instance, we have synthesized ^18^F-labeled dimeric cRGD peptide by using the condensation reaction between CBT and *N*-terminal cysteine (Table 4, entry 1). This method provided an efficient radiochemical result. However, the hydrophobic adduct produced by the radiolabeling reaction afforded high uptake values in normal organs, including in liver and kidneys compared with [^18^F]FPPRGD_2_, which is a clinically approved radiopharmaceutical [122]. Such undesired biodistribution results would hamper further investigation of new radiotracers. Therefore, development of fine-tuned functional group pairs, which are smaller and less lipophilic, and at the same time possess high reactivity and selectivity must be investigated to maximize specific targeting ability of the radioligand with minimal background signal. Consequently, bioorthogonal click reactions have exhibited enormous potential for development of radiopharmaceuticals and applications in the field of nuclear medicine. The optimization of these ligation methods will enable the exploration of advanced theranostic strategies as well as the investigation of sophisticated biological phenomena. We expect that these tools will continue to be used as a key technology for the development of various radiolabeled molecules and radiopharmaceuticals, which can offer benefits across preclinical studies and ultimately in clinical applications in the future.

## Figures and Tables

**Table 1:** Examples of SPAAC in labeling reactions using short half-life radioisotopes.

Entry	DBCO Precursor	Azide Precursor	Product ^a^	RCY(%)	Ref
1	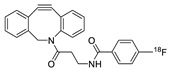	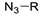 R = 4-azidoaniline, 11-azido-3,6,9-trioxaundane-1-amine, 6-azido-6-deoxyglucose, 2-azido-deoxyglucose, azido-geldanamycin	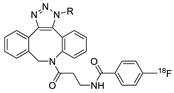	69–98	[55]
2	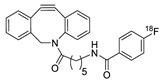	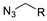 R = Ph, PEGylated acid	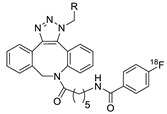	64–75	[56]
3	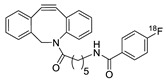	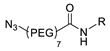 R = A20FMDV2 peptide	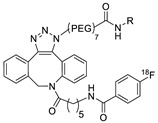	12	[57]
4	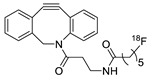	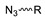 R = Tyr^3^-octreotate peptide	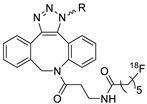	95	[58]
5	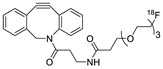	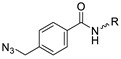 R = cRGD peptide	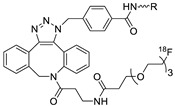	93	[59]
6	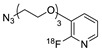	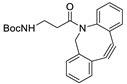	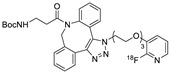	>95	[60]
7	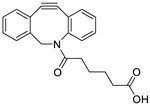	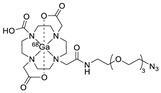 R = (PEG)_3_-DOTA-^68^Ga	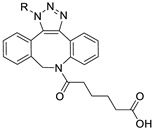	94–100	[61]
8	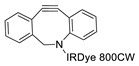	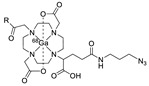 R = Tyr^3^-octreotate peptide	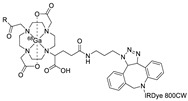	80	[62]

^a^ Products were obtained as isomeric mixtures.

**Table 2:** IEDDA-mediated in vitro radiolabeling.

Entry	Tetrazine	Dienophile	Product ^a^	RCY (%)	Ref
1	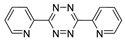	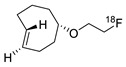	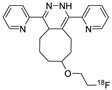	>98	[82]
2	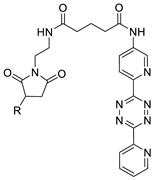 R = c(RGDyC) or VEGF protein	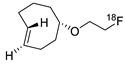	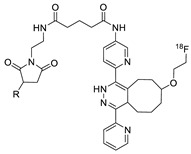	95 c(RGDyC), 75 (VEGF)	[84]
3	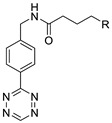 R= AZD2281	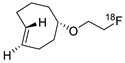	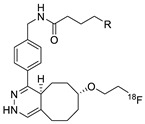	92	[85]
4	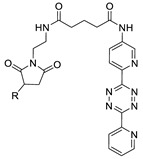 R= Cys40-exendin-4	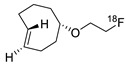	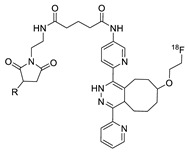	>80	[86]
5	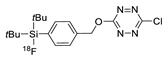	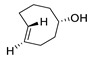	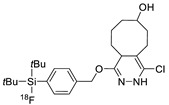	>99	[87]
6	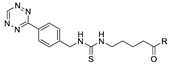 R = Bombesin	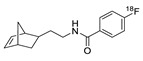	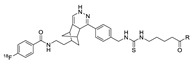	46	[88]
7	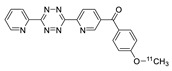	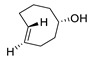	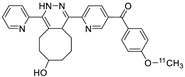	>98	[89]
8	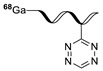 Tz-polymer	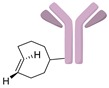 anti-A33 antibody	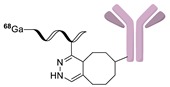	-	[90]
9	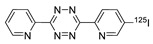	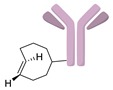 anti-VEGFR2 antibody	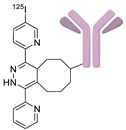	69	[91]
10	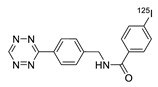	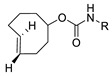 R = cRGD peptide, HSA protein	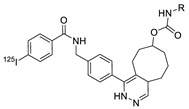	>99 (cRGD), 93 (HSA)	[92]

^a^ Products were obtained as isomeric mixtures.

**Table 3:** IEDDA-based in vivo pre-targeted approach.

Entry	Biomolecule	Radiotracer	Animal Model	Ref
1	CC49-TCO antibody	^111^In-labeled tetrazine	LS174T cells (Balb/C mouse)	[98]
2	CC49-TCO antibody	^111^In-labeled tetrazine	LS174T cells (Balb/C mouse)	[99]
3	CC49-TCO antibody	^177^Lu-labeled tetrazine	LS174T cells (Balb/C mouse)	[100]
4	CC49-TCO antibody	^177^Lu-labeled tetrazine	LS174T cells (Balb/C mouse)	[101]
5	AVP04-07-TCO diabody	^177^Lu-labeled tetrazine	LS174T cells (Balb/C mouse)	[102]
6	Z_2395_-TCO affibody	^111^In-labeled tetrazine ^177^Lu-labeled tetrazine	SKOV-3 cells (Balb/C mouse)	[103]
7	PEGylated-TCO	^18^F-labeled tetrazine	Healthy Balb/C mouse	[104]
8	5B1-TCO antibody	^18^F-labeled tetrazine	BxPC3 cells (athymic nude mice)	[105]
9	Cetuximab-TCO antibody Trastuzumab-TCO antibody	^18^F-labeled tetrazine	A431 cells (nu/nu mouse) BT-474 cells (nu/nu mouse)	[106]
10	Porous silicon-TCO nanoparticle	^18^F-labeled tetrazine	Healthy (Balb/C mouse)	[107]
11	PSMA antagonist-tetrazine conjugate	^18^F-labeled TCO	LNCaP cells (Balb/C mouse)	[108]
12	Trastuzumab-tetrazine antibody	^18^F-labeled TCO	SKOV-3 cells (Balb/C mouse)	[109]
13	A33-TCO antibody	^18^F-labeled tetrazine	LS174T cells (Balb/C mouse) A431 cells (Balb/C mouse)	[110]
14	Mesoporous silica-TCO nanoparticle	^11^C-labeled tetrazine	Healthy Balb/C mouse	[111]
15	Polyglutamic acid-TCO	^11^C-labeled tetrazine	CT26 cell (Balb/C mouse)	[112]
16	A33-TCO antibody	^64^Cu-labeled tetrazine	SW1222 cell (mouse)	[113]
17	HuA33-TCO antibody	^64^Cu-labeled tetrazine	SW1222 cell (mouse)	[114]
18	5B1-TCO antibody	^64^Cu-labeled tetrazine	BxPC3 and Capan-2 cells (athymic nude mice)	[115]
19	HuA33-dye-800-TCO	^64^Cu-labeled tetrazine	SW1222 cell (mouse)	[116]
20	C225-TCO antibody	^68^Ga-labeled tetrazine	A431 cells (Balb/C mouse)	[117]
21	HuA33-TCO antibody	^68^Ga-labeled tetrazine	SW1222 cell (CrTac:NCr- Foxn1^nu^ mouse)	[118]
22	Bisphosphonate-TCO conjugate	^177^Lu-labeled tetrazine ^99m^Tc-labeled tetrazine	Healthy Balb/C mouse	[119]
23	Bevacizumab-TCO antibody	^99m^Tc-labeled tetrazine	B16-F10 cell (C57 Bl/6J mouse)	[120]
24	CC49-TCO antibody	^212^Pb-labeled tetrazine	LS174T cells (Balb/C mouse)	[121]

**Table 4:** Aromatic prosthetic groups for radiolabeling reactions.

Entry	Radiotracer	Target Molecule	Product	RCY (%)	Ref
1	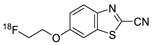	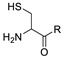 R = cRGD_2_ peptide, RLuc8 protein, Z_EGFR:1907_ affibody	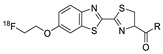	80 (cRGD), 12 (RLuc8), 41 (Z_EGFR:1907_)	[122,123]
2	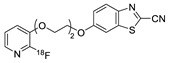	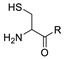 R = cRGD peptide	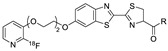	7	[124]
3	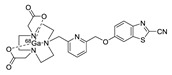	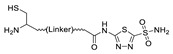	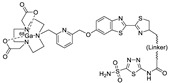	99	[125]
4	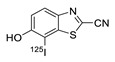	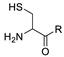 R = cRGD peptide	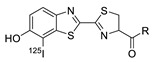	>99	[127]
5	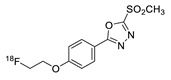	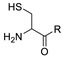 R= BBN peptide, Z_HER2:2395_ affibody	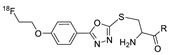	>99 (BBN), 40 (Z_HER2:2395_)	[129]
6	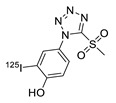	HSA protein, GCQRPPR peptide	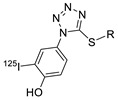 R = HSA protein, GCQRPPR peptide	65 (HSA), 99 (GCQRPPR)	[130]
7	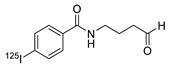	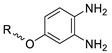 R = cRGD peptide, HSA protein	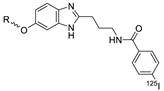	99 (cRGD), 94 (HSA)	[131]
8	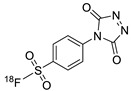	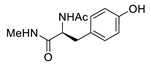	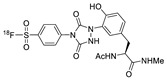	45	[132]

## Abbreviations

CBT	2-cyanobenzothiazole
CuAAC	copper(I)-catalyzed azide-alkyne [3+2] cycloaddition reaction
DBCO	dibenzocyclooctyne
DFO	deferoxamine
DMF	*N*,*N*-dimethylformamide
DMSO	dimethyl sulfoxide
DOTA	1,4,7,10-tetraazacyclododecane-tetraacetic acid
DTPA	diethylenetriaminepentaacetic acid
FH	feraheme
HPLC	high-performance liquid chromatography
HAS	human serum albumin
ICAM-1	intercellular adhesion molecule
IEDDA	inverse-electron-demand Diels–Alder reaction
MSTP	(4-(5-methane-sulfonyl-[1,2,3,4]tetrazole-1-yl)-phenol)
NOTA	1,4,7-triazacyclononane-1,4,7-triacetic acid
ODIBO	oxa-dibenzocyclooctyne
PBS	phosphate-buffered saline
PECAM-1	platelet-endothelial cell adhesion molecule
PET	positron emission tomography
PIC	polyisocyanopeptide
RCY	radiochemical yield
SPAAC	strain-promoted azide-alkyne cycloaddition reaction
SPECT	single-photon emission computed tomography
TCO	trans-cyclooctene
